# A Network Pharmacology and Experimental Validation Strategy for Hypoglycemic Study of Lonicerae Japonicae Flos on Diabetes

**DOI:** 10.1111/jcmm.71167

**Published:** 2026-05-02

**Authors:** Zhang Nan, Liu Xiaolong, Li Chunyan, Ren Lu, Guo Chao, Xue Jintao

**Affiliations:** ^1^ School of Pharmacy Henan Medical University Xinxiang Henan Province China; ^2^ Experimental Teaching Center of Biology and Basic Medicine North Henan Medical University Xinxiang Henan Province China

**Keywords:** active ingredient, diabetes, Lonicerae Japonicae Flos, molecular docking, network pharmacology, target

## Abstract

As diabetes has become an important public health issue, Lonicerae Japonicae Flos (LJF) had gradually attracted increasing attention on diabetes. This study aimed to investigate the hypoglycemic ingredients and underlying mechanisms of LJF in diabetes using an integrated strategy of network pharmacology, molecular docking and experimental validation. Network pharmacology screened 73 active ingredients, 65 targets and 20 main signalling pathways. The results of molecular docking, protein–protein interaction and ingredient‐target‐pathway network indicated that four targets (PPARG, PPARD, MGAM, GAA) and three signalling pathways (PPAR signalling pathway, Starch and sucrose metabolism, Galactose metabolism) played essential roles. Subsequently, animal experiments and in vitro tests were performed to evaluate the anti‐diabetic effect, antioxidant capacity and α‐amylase inhibitory activity for the LJF extract. Animal studies indicated the LJF alcohol extract could significantly decrease blood glucose level and mitigate the weight loss compared to the model group (*p* < 0.05), respectively. Furthermore, both in vitro and in vivo tests confirmed that the LJF alcohol extract exhibited strong antioxidant capacity (*p* < 0.05 for MDA and SOD) and α‐amylase inhibitory activity (maximum efficacy: 66.6% ± 4.6%). This strategy provided a promising approach to further elucidate the active ingredients and underlying mechanisms of LJF and other herbs.

**Abbreviations**
ABTS: 2,2′‐azinobis‐(3‐ethylbenzthiazoline‐6‐sulfonic acid ammonium salt)
ASN: asparagine
BATMAN‐TCM: Bioinformatics Annotation Database for Molecular Mechanism of Traditional Chinese Medicine
DPPH: 2, 2‐diphenyl‐1‐picrylhydrazyl radical
FBG: fasting‐blood glucose
GAA: lysosomal alpha‐glucosidase
GLU: glutamic acid
GO: Gene Ontology
HDAC2: histone deacetylase 2
HIT: Herbal Ingredients' Targets Platform
ILE: isoleucine
KEGG: Kyoto Encyclopedia of Genes and Genomes
LJF: Lonicerae Japonicae Flos
LYS: lysine
MDA: malondialdehyde
MGAM: maltase‐glucoamylase, intestinal
NR3C2: mineralocorticoid receptor
PDB: Protein Data Bank
PPARG: peroxisome proliferator‐activated receptor gamma
PPI: protein–protein interaction
PRKAB1: 5′‐AMP‐activated protein kinase subunit beta‐1
PTGS1: prostaglandin G/H synthase 1
RAR: retinoic acid receptor
SD: standard deviation
SOD: superoxide dismutase
TCMSP: Traditional Chinese Medicine Systems Pharmacology Database and Analysis Platform
TRP: tryptophan

## 1 Introduction

As one of the most prominent global diseases currently, diabetes is characterized by chronic hyperglycemia [1]. Long‐term hyperglycemia would lead to many severe complications, such as nephropathy, liver disease, cerebrovascular disease, coronary heart disease and other vascular diseases [2, 3, 4]. In the past decades, the prevalence of diabetes had increased dramatically. According to the International Diabetes Federation, there were 463 million diabetic adults in 2019, resulting in 4.2 million deaths and more than 720 billion US dollars in health expenditures, and China ranked first with 125 million cases. Furthermore, this number is projected to reach 700 million by 2045, indicating that approximately 10% of the global population will be afflicted with diabetes [3, 4, 5].

As a major public health issue, diabetes has prompted an urgent demand for effective drugs [3, 4, 5, 6]. Natural herbs, especially traditional Chinese medicine, would be an effective therapeutic method. With centuries of practical applications on hyperglycemia, traditional Chinese medicine had accumulated enormous experience in control of high blood glucose. The multi‐ingredient and multi‐target characteristics of traditional Chinese medicine played an essential role in its therapeutic efficacy. However, these features also presented considerable difficulties in elucidating its active ingredients and mechanisms on diabetes [1].

Characterized by the systematic and holistic feature, network pharmacology is consistent with the overall concept and dialectical therapeutic principle of traditional Chinese medicine [5]. With the advantages of bioinformatics methodology and network topology, network pharmacology gradually becomes a promising approach to deeply screen the potential active ingredients, targets and molecular mechanisms for traditional Chinese medicine [2, 7, 8]. In addition, molecular docking method could be employed to evaluate the binding interactions between target and active ingredient [9]. Yin Bei et al. used network pharmacology and molecular docking analysis to study the active components and mechanisms of Huanglian Jiedu Decoction on diabetes [5]. Su Lili et al. found three active ingredients of Danggui Buxue Decoction could improve diabetic nephropathy through network pharmacology and experimental validation [2]. Based on our previous researches, network pharmacology had been used to screen the active ingredients and mechanisms for Isodon Japonicus [10], Scutellariae Radix [11] and Lian‐Ge granules [12].

LJF, named Jin‐Yin‐Hua in Chinese, are the buds and newly bloomed flowers of *Lonicera japonica* Thunb [13, 14, 15, 16]. With a history spanning nearly two millennia, LJF had been used as a well‐known edible‐functional food with many beneficial properties [15, 16, 17]. Modern pharmacological studies revealed that LJF possesses broad biological activities including anti‐inflammatory, antibacterial, antiviral, antioxidant, hypoglycemic effect and lowering lipidemia activity [6, 13, 15, 16, 17, 18, 19, 20]. Recent studies had increasingly focused on the hypoglycemic effect of LJF and identification of its bioactive ingredients. Guo C. et al. studied the anti‐diabetic effect of the LJF extract and its bioactive ingredient (chlorogenic acid) [19]. Liu Z. et al. isolated two compounds from LJF, Lonjaponspiroside A and B, both of which showed good inhibitory effect against α‐glucosidase and protein tyrosine phosphatase 1B [20]. Although the hypoglycemic effect of LJF has been demonstrated, its active ingredients and underlying pharmacological mechanisms remain unclear. To the best of our knowledge, no systematic study has yet been conducted to date on the active constituents, targets and mechanisms of LJF in diabetes.

In this study, network pharmacology was firstly employed to comprehensively screen the active ingredients, potential targets and signalling pathways for LJF on diabetes. The predicted findings were subsequently validated through molecular docking, enzyme activity and antioxidant activity experiments. In parallel, a comparative study was conducted to evaluate the hypoglycemic effects of the alcohol extract and water extracts of LJF, providing a scientific basis for future research on LJF.

## 2 Materials and Methods

### 2.1 Chemicals and Reagents

Streptozotocin (No. RH222010, WXBC5204V) was obtained from Shanghai Rhawn Chemical Technology Co. Ltd. (Shanghai, China) and Sigma‐Aldrich Company (Saint Louis, Missouri, USA). Metformin (No. H11021508) was purchased from Beijing Jingfeng Pharmaceutical Co. Ltd. (Beijign, China). The glucometer (No. 586) was the product of Yuyue Kailite Biotechnology Co. Ltd. (Jiangsu, China). Total superoxide dismutase (SOD) assay kit (No. A001‐1‐1) and malondialdehyde (MDA) assay kit (No. A003‐1‐2) were from Nanjing Jiancheng Bioengineering Institute (Nanjing, China). 2, 2′‐azinobis‐(3‐ethylbenzthiazoline‐6‐sulfonic acid ammonium salt) (ABTS, No. G2107021), 2, 2‐diphenyl‐1‐picrylhydrazyl radical (DPPH, No. B2222178) and α‐amylase (No. A109181) were supplied by Beijing InnoChem Science & Technology Co. Ltd. (Beijing, China). Water was purified twice. All other reagents were of analytical grade.

### 2.2 Preparation of Plant Material

The plant name of LJF had been checked with Chinese Pharmacopoeia (2020 Edition, Volume 1) and The Plant List (http://www.theplantlist.org). Our institute is located in the authenticated region of LJF (Fengqiu County, Xinxiang City, Henan Province). With this advantage, the LJF samples with excellent quality were collected. The LJF sample collection process was followed the Convention on Biological Diversity and the Nagoya Protocol. The samples were authenticated by Xue Jintao in Jun. 2021. The voucher specimens were deposited in the School of Pharmacy, Henan Medical University, Xinxiang, China.

### 2.3 LJF Alcohol Extract

Ten gram of the samples were heated to reflux with 100 mL of 95% ethanol for 1 h. After two cycles, the extraction solution was combined, filtered and condensed to the appropriate volume without ethanol by rotary evaporation. The LJF alcohol extract (0.1 g/mL) was prepared as liquid and stored at 4°C. The dosage of alcohol extract (1.39 g/kg/day for animal experiment) was calculated based on the crude LJF.

### 2.4 LJF Water Extract

10 g of the samples were decocted with 100 mL of water for 1 h in two times. Then, the mixture extraction solution was processed with the same method of alcohol extract. The LJF water extract (0.1 g/mL) was prepared as liquid and stored at 4°C. The dosage of water extract (1.39 g/kg/day for animal experiment) was calculated based on the crude LJF.

### 2.5 Quality Analysis of LJF

Three batches of samples (No. B20190515, C20190520 and D20190520) were analysed according to the methods of Chinese Pharmacopoeia (2020 Edition, Volume 1) and previous study [14]. The contents of chlorogenic acid (5‐O‐caffeoylquinic acid), isochlorogenic acid A (3,5‐dicaffeoylquinic acid) and isochlorogenic acid C (4,5‐dicaffeoylquinic acid) were determined with Metage QuasIR (Vspec, USA) according to the method in our previous study [14]. The water content, the total ash content and the acid insoluble ash content were measured according to the method described in the LJF monograph of the Chinese Pharmacopoeia.

### 2.6 Network Pharmacology

The LJF active ingredients were retrieved from the Traditional Chinese Medicine Systems Pharmacology Database and Analysis Platform (TCMSP) database (Version 2.3, https://www.tcmsp‐e.com/), the Herbal Ingredients' Targets Platform (HIT) 2.0 database (http://www.badd‐cao.net:2345/) and the literatures as shown in Figure S1 [16, 17, 20, 21, 22]. Then, the chemical structures of the active ingredients were drawn with ChemDraw software (Version 14.0, PerkinElmer Co. Ltd.), and uploaded to the Bioinformatics Annotation Database for Molecular Mechanism of Traditional Chinese Medicine (BATMAN‐TCM) database (http://bionet.ncpsb.org.cn/batman‐tcm/) and the Swiss Target Prediction database (http://www.swisstargetprediction.ch/) in appropriate formats changed by Open Bable software (Version 2.4.1). Then, the targets were screened with the Z'‐score more than 23.0 for the BATMAN‐TCM database and the probability more than 0 for the Swiss Target Prediction database. With the keywords ‘diabetes’, the diabetic targets in *Homo sapiens* , the diabetic targets were summarized from the DRUGBANK database (https://go.drugbank.com/), the GeneCard database (Version 5.14, https://www.genecards.org/) and the UniProt database (https://www.uniprot.org/). For the GeneCard database, the scoring thresholds was set at 10. At last, the hypoglycemic ingredients and targets of LJF were screened through the intersection analysis between the targets of ingredients and the diabetic targets.

The hypoglycemic targets of LJF were uploaded to the String database (version 11.5, https://cn.string‐db.org/) for the analysis of the protein–protein interaction (PPI) to select the core hypoglycemic targets. Then, the core hypoglycemic targets were used for Gene Ontology (GO) and the Kyoto Encyclopedia of Genes and Genomes (KEGG) enrichment analysis via the Metascape database (https://metascape.org/). Finally, an ingredient‐target‐pathway network for LJF was constructed by Cytoscape software (Version 3.9.0).

### 2.7 Molecular Docking

For computational preparation of ligand structures, the two‐dimensional chemical configuration of bioactive compounds was set energy minimization through molecular mechanics optimization in ChemBio3D Ultra 14.0 software (CambridgeSoft, Cambridge, MA, USA), employing thermodynamic principles to achieve minimal free energy conformation. The target protein was retrieved from the Protein Data Bank (PDB) database (https://www.rcsb.org/). Then this computational protocol systematically executed hydrogen atom incorporation, electrostatic charge parameterization, and protonation state refinement to achieve biophysically relevant receptor configurations by the Autodock Tools software (Version 1.5.6, Scripps Research Institute). The resultant energy‐minimized macromolecular structure was subsequently integrated into the docking simulations as the target binding platform. With other parameters set at default values, the Autodock Vina software (Version 1.1.2, Scripps Research Institute) was used to perform the semiflexible docking and calculate the affinity between the target and ingredient. The visualization of the docking result was adjusted at appropriate angles by the PyMoL software (Version 1.7.2.1, Schrodinger Inc.) [5, 8, 9, 20].

### 2.8 Animal Experiment

Sprague Dawley healthy rats (8 weeks, male, *n* = 36) were obtained from the Laboratory Animal Center of Henan Medical University (Henan, China). After acclimatization in the standard environment for 1 week, 30 rats (diabetic model rats) were randomly assigned high‐sugar and high‐fat diets (10% sucrose, 10% lard and 2% NaCl added to the normal diet), with an energy composition of approximately 65% carbohydrate, 24% fat and 11% protein. The remaining six rats (control non‐diabetic group) were fed regular diets (energy composition: 75% carbohydrate, 10% fat, 15% protein) which were got from the Laboratory Animal Center of Henan Medical University (Henan, China). Two weeks later, the diabetic model rats were induced with streptozotocin (45 mg/kg, single intraperitoneal injection) after 12 h of fasting [6, 23]. At the same time, the same dose of saline was given for the control non‐diabetic group. After 3 days, the rats with fasting blood glucose values ≥ 16.6 mmol/L were enrolled as diabetic model rats. At last, 26 rats were successfully induced as diabetic model rats, and 24 diabetic model rats were randomly selected for the following study.

The diabetic model rats were randomly distributed into 4 groups (*n* = 6) and administrated once daily by gavage for 6 weeks: (1) Model group, receiving purified water; (2) Positive group, receiving metformin (0.16 g/kg/day) [24]; (3) Water extract group, receiving the LJF water extract (1.39 g/kg/day); (4) Alcohol extract group, receiving the LJF alcohol extract (1.39 g/kg/day) [6, 19]. Control non‐diabetic group (*n* = 6) was administrated with the same dose of water. The dosage of LJF extract was set according to the Chinese Pharmacopoeia (2020 Edition, Volume 1) and the literatures [6, 19]. Based on the dose of the Chinese Pharmacopoeia (2020 Edition, Volume 1) for adult, the dosage of our study was calculated for rats. During the treatment period, the body weight and blood glucose were monitored weekly. At last, the rats were anaesthetised with isoflurane [25]. Then, the blood samples were got from the abdominal aorta for the subsequent study, and the tissues of the pancreas were collected for haematoxylin and eosin staining [2, 10, 19]. At last, the rats were euthanized by cervical dislocation in accordance with institutional ethical guidelines [1, 2, 6, 19].

The above animal experiment was approved by the Animal Ethics Committee of Henan Medical University (No. XYLL‐20220169) and followed the internationally accepted principles for laboratory animal use and care, such as the European Community guidelines (EEC Directive of 1986; 86/609/EEC) and Guidelines for the Euthanasia of Animals (2020).

### 2.9 In Vitro and In Vivo Antioxidant Activity

#### 2.9.1 Antioxidant Activity

The serum was thawed at room temperature. According to the instructions of the assay kits, a proper amount of supernatant was used for quantification. The content of SOD in serum (*n* = 6) was determined by the xanthine oxidase method, while the level of MDA in serum (*n* = 6) was analysed by the thiobarbituric acid method.

#### 2.9.2 ABTS Method

7.00 mmol/L of ABTS solution was oxidized by 2.45 mmol/L of potassium persulphate for 16 h in the dark to obtain ABTS cationic solution. Then, it was diluted to an absorbance of 0.70 ± 0.02 at 734 nm with 0.1 mmol/L of phosphate buffer (pH 7.0) with anhydrous ethanol as blank solution. 3.9 mL of the above prepared ABTS solution was mixed with 0.1 mL of the LJF water extract (2.0–6.0 mg/mL) or the alcohol extract (4.0–12.0 mg/mL). After the mixture solution incubated in the dark for 10 min at room temperature, the absorbance was measured at 734 nm. The tests were performed in triplicate [26, 27].

#### 2.9.3 DPPH Method

With 1.0 mL of anhydrous ethanol as control solution, 1.0 mL of the LJF water extract (0.05–0.40 mg/mL) or the LJF alcohol extract (0.30–2.70 mg/mL) was added to 3.0 mL of the DPPH solution (0.2 mmol/L) for 30 min in the dark. Then, the absorbance was measured at 517 nm. The tests were performed in triplicate [26, 27].

### 2.10 α‐Amylase Inhibitory Effect

0.25 mL of the α‐amylase solution (1.5 U/mL) was mixed with 0.25 mL of the LJF water extract (50–90 mg/mL) or the LJF alcohol extract (100–200 mg/mL), and incubated in a water bath at 37°C for 10 min. With purified water as the blank solution, the reaction was started by adding 0.5 mL of 1% starch solution (w/v) at 37°C for 10 min. Then, the reaction was terminated by adding 1 mL of 3,5‐dinitrosalicylic acid solution in a boiled water bath for 10 min. After cooling to room temperature in the dark, 10.0 mL purified water was mixed with the above solution. With absorbance measured at 540 nm, the inhibitory rate of α‐amylase (%) was calculated by the equation: the inhibitory rate = [1 − (*A* − *B*)/*B*] × 100%, where *A* was the absorbance of each extract sample; *B* was the absorbance of the blank group. The tests were performed in triplicate [6, 20, 28].

### 2.11 Statistical Analysis

The data are expressed as mean ± standard error. Comparisons between groups were made by one‐way ANOVA of the SPSS 13.0 software (SPSS Inc., Chicago, IL, USA). A *p* < 0.05 means there is a statistically significant difference.

## 3 Results

### 3.1 Hypoglycemic Ingredients, Targets and PPI Network

**FIGURE 1:**
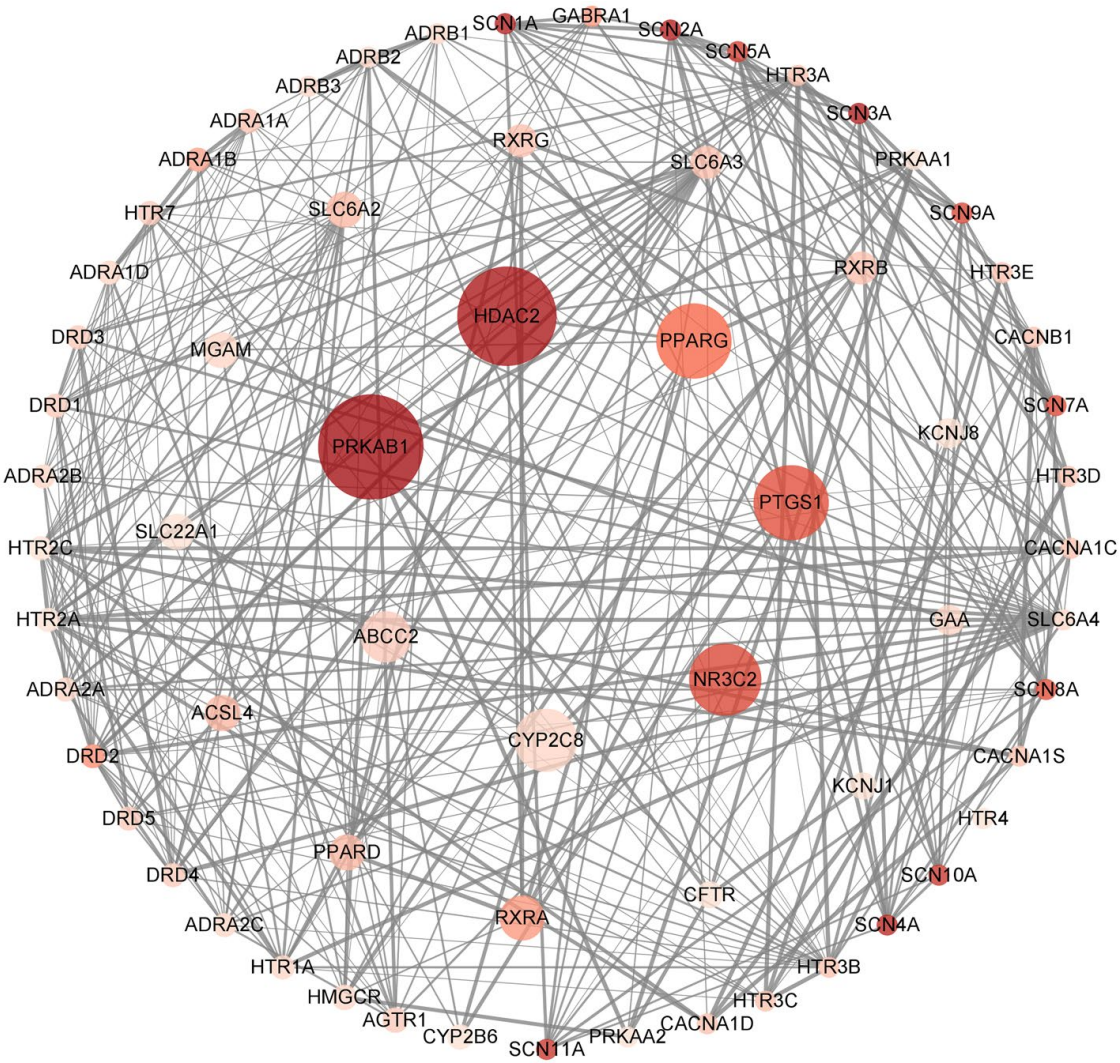
The protein–protein interaction (PPI) of hypoglycemic targets for Lonicerae Japonicae Flos (the node represented the target, while the edge indicated there was an association between the two targets; the size of each target node was determined by the relevance score; the intensity of colour represents the number of ingredients; the width of the edge was for combined score of PPI).

A total of 186 original chemical constituents of LJF (Table S1) were obtained from the TCMSP database, the HIT2.0 databases and the literature [16, 17, 20, 21, 22]. As shown in Figure S2, 535 corresponding targets of those ingredients were retrieved from the BATMAN‐TCM database and the Swiss Target Prediction database. An intersection analysis with 195 diabetes‐related targets from the DrugBank database yielded 65 potential hypoglycemic targets for LJF (Figure 1). Subsequently, a PPI network comprising 65 nodes and 275 edges was constructed using the String database (Figure 1). In the PPI network, the nodes represented the hypoglycemic targets, and the edges illustrated there was an association between the intersectional targets. The targets which were closely related would play the same or synergistic role in the disease prevention and control. According to the topology analysis for the PPI network, the average local clustering coefficient was 0.603 with 8.46 as the average node degree. Based on the scoring system comprising relevance scores, PPI network parameters and the number of associated ingredients, 20 core hypoglycemic targets of LJF were identified (Table 1). A total of 73 active ingredients from LJF were found to be related to these 20 core targets (Table S1). Our findings suggested that the hypoglycemic effects of LJF might be achieved through coordinated regulation of multiple key targets, including 5′‐AMP‐activated protein kinase subunit beta‐1 (PRKAB1), histone deacetylase 2 (HDAC2), prostaglandin G/H synthase 1 (PTGS1), mineralocorticoid receptor (NR3C2) and peroxisome proliferator‐activated receptor gamma (PPARG), as well as possibly others (Figure 1 and Table 1). These targets exhibited concerted modulation effects of LJF on diabetes.

**TABLE 1:** Information of the core hypoglycemic targets for Lonicerae Japonicae Flos.

Target gene	Uniport ID	Target	Number of ingredients	Primary KEGG signalling pathways
PRKAB1	Q9Y478	5′‐AMP‐activated protein kinase subunit beta‐1	24	Adipocytokine signalling pathway; AMPK signalling pathway
HDAC2	Q92769	Histone deacetylase 2	21	/
PTGS1	P23219	Prostaglandin G/H synthase 1	16	Arachidonic acid metabolism
NR3C2	P08235	Mineralocorticoid receptor	15	/
PPARG	P37231	Peroxisome proliferator‐activated receptor gamma	13	PPAR signalling pathway; AMPK signalling pathway
RXRA	P19793	Retinoic acid receptor RXR‐alpha	7	PPAR signalling pathway; Adipocytokine signalling pathway; Th17 cell differentiation
ACSL4	O60488	Long‐chain‐fatty‐acid–CoA ligase 4	5	PPAR signalling pathway; Adipocytokine signalling pathway
PPARD	Q03181	Peroxisome proliferator‐activated receptor delta	5	PPAR signalling pathway
SLC6A2	P23975	Sodium‐dependent noradrenaline transporter	5	/
RXRB	P28702	Retinoic acid receptor RXR‐beta	4	PPAR signalling pathway; Adipocytokine signalling pathway; Th17 cell differentiation
RXRG	P48443	Retinoic acid receptor RXR‐gamma	4	PPAR signalling pathway; Adipocytokine signalling pathway; Th17 cell differentiation
SLC6A3	Q01959	Sodium‐dependent dopamine transporter	4	/
ABCC2	Q92887	Canalicular multispecific organic anion transporter 1	3	ABC transporters
GAA	P10253	Lysosomal alpha‐glucosidase	2	Galactose metabolism; Starch and sucrose metabolism
MGAM	O43451	Maltase‐glucoamylase, intestinal	2	Galactose metabolism; Starch and sucrose metabolism
CYP2C8	P10632	Cytochrome P450 2C8	1	Arachidonic acid metabolism
CFTR	P13569	Cystic fibrosis transmembrane conductance regulator	1	AMPK signalling pathway; ABC transporters
KCNJ1	P48048	ATP‐sensitive inward rectifier potassium channel 1	1	/
KCNJ8	Q15842	ATP‐sensitive inward rectifier potassium channel 8	1	/
SLC22A1	O15245	Solute carrier family 22 member 1	1	/

### 3.2 GO and KEGG Enrichment Analysis

**FIGURE 2:**
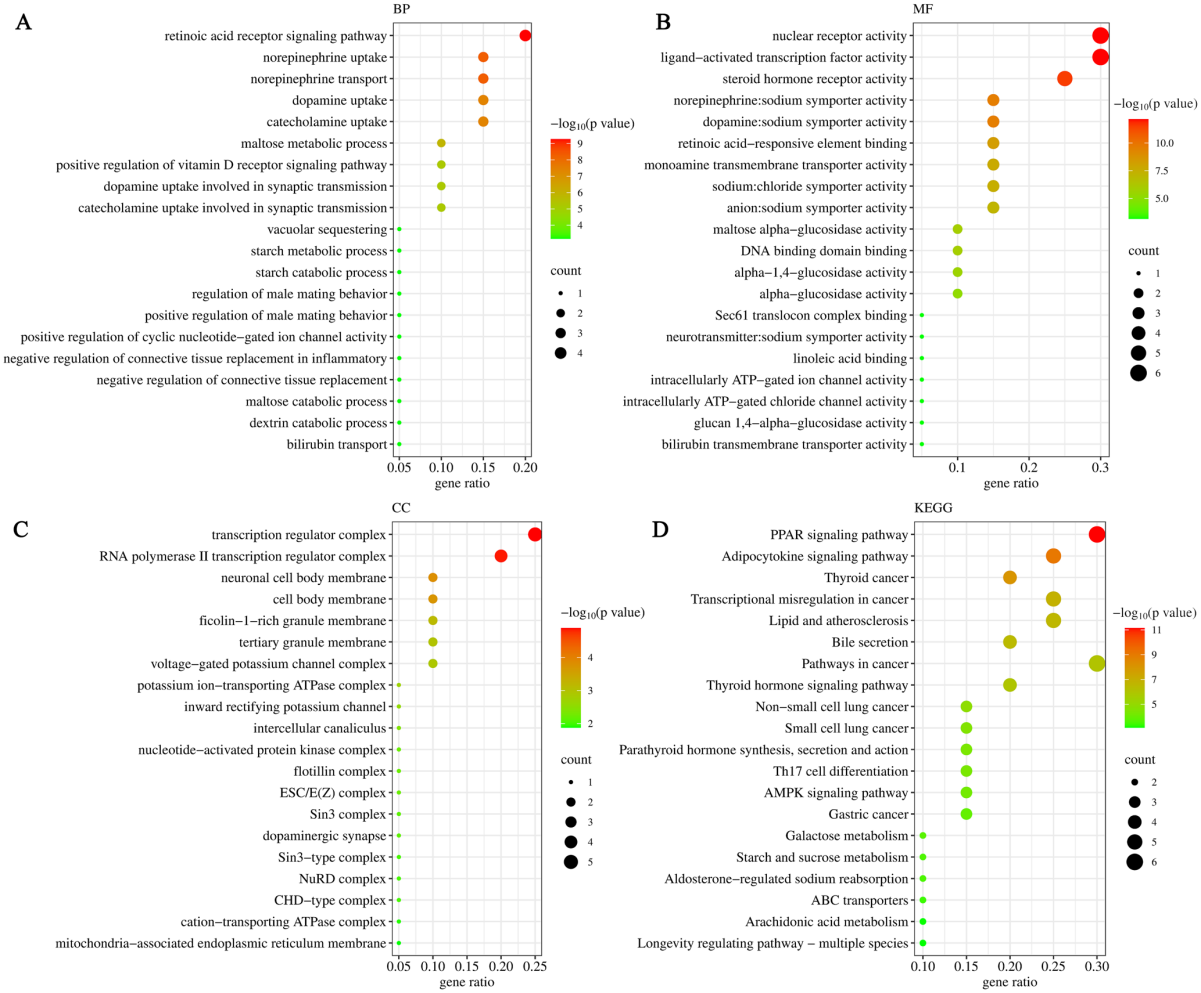
The Gene Ontology enrichment analysis (A—biological processes, B—molecular functions and C—cellular components) and the Kyoto Encyclopedia of Genes and Genomes (KEGG) enrichment analysis (D): The horizontal coordinate showed the values of gene ratio; the size of the circle meant the number of related targets; the colour of the circle indicated the value of the significance (*p*).

The GO enrichment analysis was summarized through three modules: biological process, molecular function and cellular component. As revealed in Figure 2 and Table S2, GO enrichment analysis was performed based on the above 20 core hypoglycemic targets. According to the *p* value, the count of targets and the gene ratio, the main biological processes (Figure 2A) for the hypoglycemic effect included retinoic acid receptor (RAR) signalling pathway, maltose metabolic process, starch metabolic process and so on. As shown in Figure 2B, the molecular functions mainly involved maltose alpha‐glucosidase activity, alpha‐1,4‐glucosidase activity, alpha‐glucosidase activity, etc. As listed in Figure 2C, the main cellular components were transcription regulator complex, RNA polymerase II transcription regulator complex, voltage‐gated potassium channel complex and so on.

The KEGG enrichment analysis is an efficient method for screening the signalling pathways. Based on *p* value, count of targets and gene ratio, the 20 primary KEGG signalling pathways were depicted in Figure 2D. These pathways, including the PPAR signalling pathway, Adipocytokine signalling pathway, Galactose metabolism and Starch and sucrose metabolism, may play a crucial role in the hypoglycemic effect of the LJF active ingredients.

### 3.3 Hypoglycemic Effect on Diabetic Rats

#### 3.3.1 Quality Analysis

As shown in Table 2, the LJF quality was analysed according to the methods of Chinese Pharmacopoeia (2020 Edition, Volume 1) and previous study [14]. So, the samples used in this study had outstanding quality and met the standards of Chinese Pharmacopoeia.

**TABLE 2:** Quality analysis of Lonicerae Japonicae Flos (mean ± standard error, *n* = 3).

	Experimental value (%)	Limits of Chinese Pharmacopoeia (%)
Chlorogenic acid	3.203 ± 0.595	≥ 1.5 for chlorogenic acid; ≥ 3.8 for the sum of chlorogenic acid, isochlorogenic acid A and isochlorogenic acid C
Isochlorogenic acid A	1.454 ± 0.396	
Isochlorogenic acid C	0.249 ± 0.033	
Water content	10.16 ± 1.28	≤ 12.0
Total ash content	6.14 ± 0.31	≤ 10.0
Acid insoluble ash content	0.25 ± 0.11	≤ 3.0

#### 3.3.2 Changes in Blood Glucose and Pancreas

**FIGURE 3:**
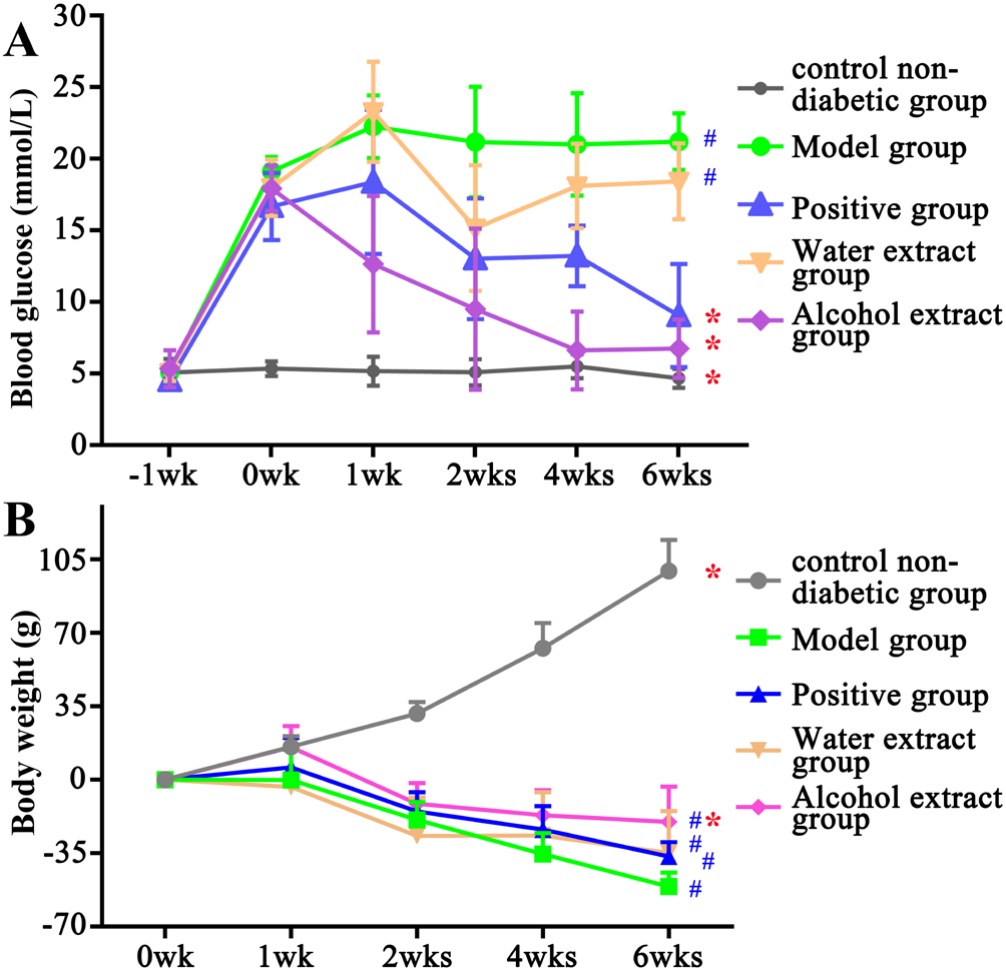
The blood glucose level (A) and body weight changes (B) of each group (mean ± standard error; ‘‐1wk’ means the blood glucose level before inducing to diabetic model, and ‘0wk’ means the blood glucose level after 3 days of inducing to diabetic model. ‘wk(s)’ means the blood glucose level and body weight change of each week after the treatment). Data are expressed as mean ± SD, *n* = 6. * *p* < 0.05 versus model group; ^#^ *p* < 0.05 versus control non‐diabetic group; Comparisons between groups were made by one‐way ANOVA.

The β‐cells in the pancreas which secrete insulin are responsible for the control of blood glucose, so an impaired or loss of islet cells would primarily increase the level of blood glucose [3]. As revealed in Figure S3, the pancreatic islets of the control non‐diabetic group were in round shape with clear nuclei, and arranged regularly and densely. In the diabetic group, the islets were irregularly shaped, loosely arranged and sparsely distributed. As shown in Figure 3, there were no significant differences in the blood glucose level of each group before inducing the diabetic model (*p* > 0.05), respectively. However, after inducing the diabetic model, the blood glucose levels of diabetic model rats were significantly increased compared to the control non‐diabetic group (*p* < 0.05, 17.94 ± 1.89 mmol/L vs. 5.35 ± 0.52 mmol/L). The alterations in blood glucose levels and islets indicated that the diabetic model rats were successfully induced for the following study. After 6 weeks of treatment as shown in Figure 3A, the blood glucose levels of the alcohol extract group and the positive group were significantly decreased compared with the model group (*p* < 0.05, 6.75 ± 2.03 mmol/L vs. 21.22 ± 1.98 mmol/L), respectively. In addition, there was no significant difference between the alcohol extract group and the positive group (*p* > 0.05, 6.75 ± 2.03 mmol/L vs. 9.07 ± 3.61 mmol/L).

#### 3.3.3 Changes in Weight

According to the World Health Organization, energy supply deficiency leads to protein and fat consumption, making weight loss a major symptom of diabetes [2, 3, 4]. As shown in Figure 3B, the body weight of the control non‐diabetic group increased gradually over 6 weeks of treatment, whereas the weights of the remaining groups decreased significantly (*p* < 0.05), respectively. Compared to the weight loss of the model group, a significant weight change (*p* < 0.05) was observed in the alcohol extract group, while that of the positive group and the water extract group had no significant difference (*p* > 0.05), respectively.

In summary, the findings of the animal experiments indicated that the LJF alcohol extract exhibited significant hypoglycemic effects and attenuated diabetes‐induced weight loss.

### 3.4 Hypoglycemic Ingredients and Mechanism

#### 3.4.1 Hypoglycemic Ingredients

In general, the solubility and cell membrane permeability of a chemical ingredient are closely associated with its log *p* value [10, 29]. The value of log *p* < 1 means the ingredient has a good water solubility. According to Table S1, only four ingredients of the 73 LJF hypoglycemic ingredients had the value of log *p* < 1, whereas the majority exhibited good ethanol solubility. Consistent with the in vivo findings, the alcohol extract of LJF exerted a marked hypoglycemic effect, whereas the water extract showed no significant activity. Thereby, these two results could be mutually corroborated. A log *p* value ranging from 0 to 3 is deemed optimal for achieving a favourable pharmacokinetic property, characterized by efficient oral absorption, balanced volume of distribution and adequate blood–brain barrier permeability [10, 29]. As shown in Table 3, 20 hypoglycemic ingredients with 0–3 of log *p* were got, and these ingredients might be the key hypoglycemic ingredients for LJF on diabetes [6, 13, 19].

**TABLE 3:** The chemical information and targets of the key hypoglycemic ingredients of Lonicerae Japonicae Flos.

Ingredients	Cas No.	AlogP	Targets
3‐penten‐2‐one	3102‐33‐8	0.86	2 (HDAC2, NR3C2)
Protocatechuic acid	99–50–3	0.90	2 (PPARG, PTGS1)
4‐hydroxybenzoic acid	99–96‐7	1.17	2 (PPARG, PTGS1)
Hydroquinone	123–31‐9	1.30	1 (RXRA)
Caffeic acid	331–39‐5	1.37	1 (PPARG)
3‐(3,4‐dihydroxyphenyl) propionic acid	1078‐61‐1	1.39	3 (PPARG, PTGS1, SLC6A2)
cis‐2‐furanmethanol	5989‐33‐3	1.43	1 (ABCC2)
Linalool oxide	1365‐19‐1	1.43	1 (ABCC2)
Phenylacetaldehyde	122–78‐1	1.52	3 (PPARG, PTGS1, CFTR)
Propyl vinyl ketone	1629–60–3	1.58	2 (HDAC2, NR3C2)
Benzaldehyde	100–52‐7	1.59	2 (PPARG, SLC6A3)
Methyl caffeate	3843‐74‐1	1.62	1 (PTGS1)
p‐hydroxycinnamic acid	7400‐08‐0	1.64	2 (PPARG, PTGS1)
Sulcatone	110–93‐0	1.79	2 (HDAC2, NR3C2)
Methyl 4‐hydroxycinnamate	3943‐97‐3	1.89	1 (PTGS1)
Indole	120–72‐9	2.12	4 (KCNJ1, KCNJ8, SLC22A1, SLC6A2)
Octanoic acid	124–07‐2	2.72	1 (HDAC2)
1‐hexene	592–41‐6	2.72	1 (PRKAB1)
1‐octanol	111–87‐5	2.80	1 (HDAC2)
(1S)‐(−)‐alpha‐pinene	7785‐26‐4	2.87	2 (NR3C2, PRKAB1)

**FIGURE 4:**
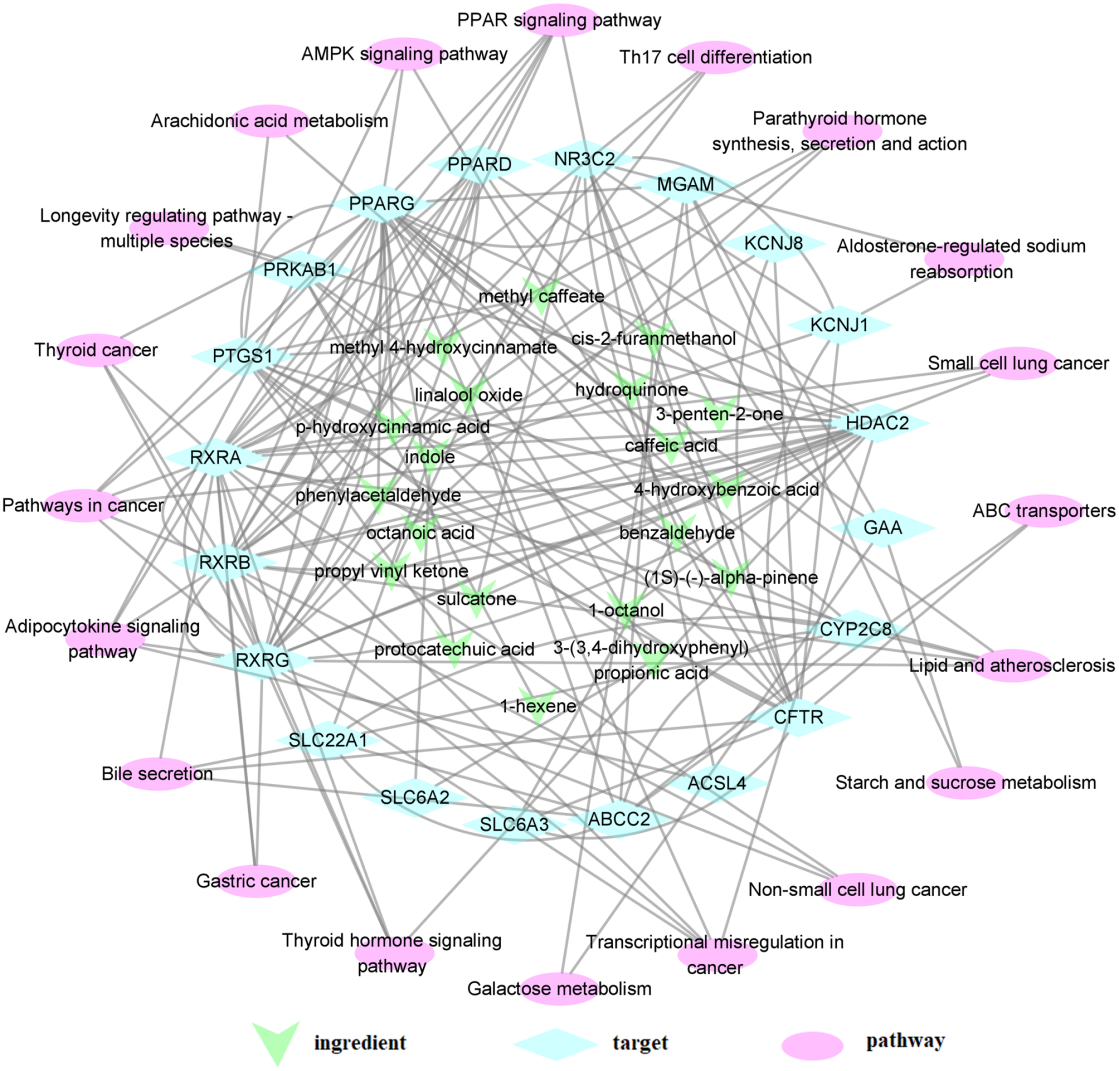
The ingredient‐target‐pathway network of the hypoglycemic effect for Lonicerae Japonicae Flos on diabetes (the node represented ingredient, target or pathway, while the edge indicated there was association between two nodes; the arrow represented the key hypoglycemic ingredients, and the diamond showed the core hypoglycemic targets with the ellipse for the primary KEGG signalling pathways).

The key hypoglycemic ingredients, the core targets and the primary KEGG signalling pathways were used to construct the ingredient‐target‐pathway network for LJF on diabetes (Figure 4). Based on the network analysis, an individual active ingredient from LJF can modulate multiple targets, which in turn are involved in diverse signalling pathways. For instance, p‐hydroxycinnamic acid was found to interact with both PPARG and PTGS1. Notably, PPARG and PTGS1 were targeted by 13 and 16 hypoglycemic ingredients of LJF, respectively, indicating potential synergistic effects. The results of GO and KEGG enrichment analysis and the ingredient‐target‐pathway network suggested that these pathways, such as PPAR signalling pathway, Galactose metabolism and Starch and sucrose metabolism, may be the main mechanism for LJF on diabetes.

#### 3.4.2 Antioxidant Activity

In this study, the antioxidant capacity was evaluated by the antioxidant assays and the molecular docking method.

**FIGURE 5:**
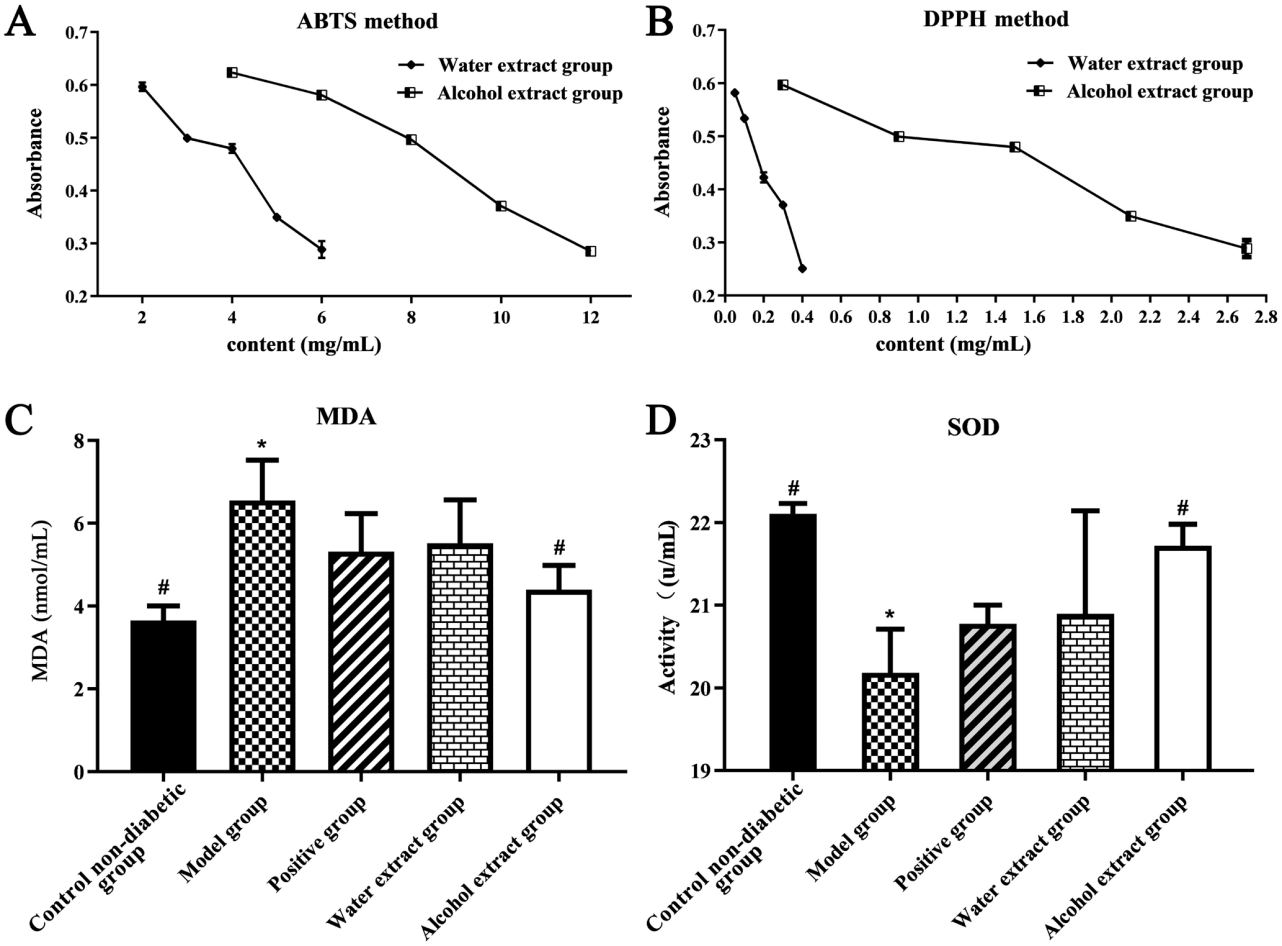
The antioxidant capacity of Lonicerae Japonicae Flos (A—the ABTS method; B—the DPPH method; C—MDA; D—SOD; mean ± standard error, *n* = 3 for A and B, and *n* = 6 for C and D; *p < 0.05 means significant difference compared to control non‐diabetic group; ^#^ *p* < 0.05 means significant difference compared to model group; Comparisons between groups were made by one‐way ANOVA).

Both the ABTS and DPPH assays are reliable methods to investigate the free radical scavenging capacity of the antioxidants in food and biological field. The total antioxidant activity was quantified by assessing the reduction of ABTS or DPPH radical cations [26, 27]. The antioxidant capacity was inversely correlated with the absorbance measured. As shown in Figure 5A,B, both the LJF water and alcohol extracts exhibited potent antioxidant activity in a dose‐dependent manner, and the water extract exerted superior radical scavenging capacity compared to the alcohol extract.

To investigate the in vivo antioxidant effect of the LJF extract, the levels of MDA and SOD were measured, which serve as markers of oxidative stress and endogenous antioxidant capacity, respectively. As demonstrated in Figure 5C,D, compared with control non‐diabetic group, the level of MDA in the model group was significantly increased (*p <* 0.05), while the level of SOD was significantly decreased (*p <* 0.05). After 6 weeks of treatment, the LJF alcoholic extract could significantly decrease the MDA level (*p* < 0.05) and improve the SOD level (*p <* 0.05) compared with the model group, respectively. Moreover, there was no significant difference between the alcoholic extract group and the control non‐diabetic group for the levels of MDA and SOD (*p* > 0.05), respectively. Despite the good antioxidant activity in vitro, the LJF water extract showed no significant in vivo antioxidant or hypoglycemic effects. This discrepancy can be partly explained by its chemical composition. As listed in Table S1, most of the 73 predicted hypoglycemic ingredients from LJF showed good ethanol solubility (log *p* > 1), and therefore preferentially distributed in the alcohol fraction. Consequently, the alcohol extract, enriched with these lipophilic compounds, exhibited potential antioxidant and hypoglycemic effects in diabetic rats, whereas the water‐soluble components appeared to lack in vivo bioavailability.

**FIGURE 6:**
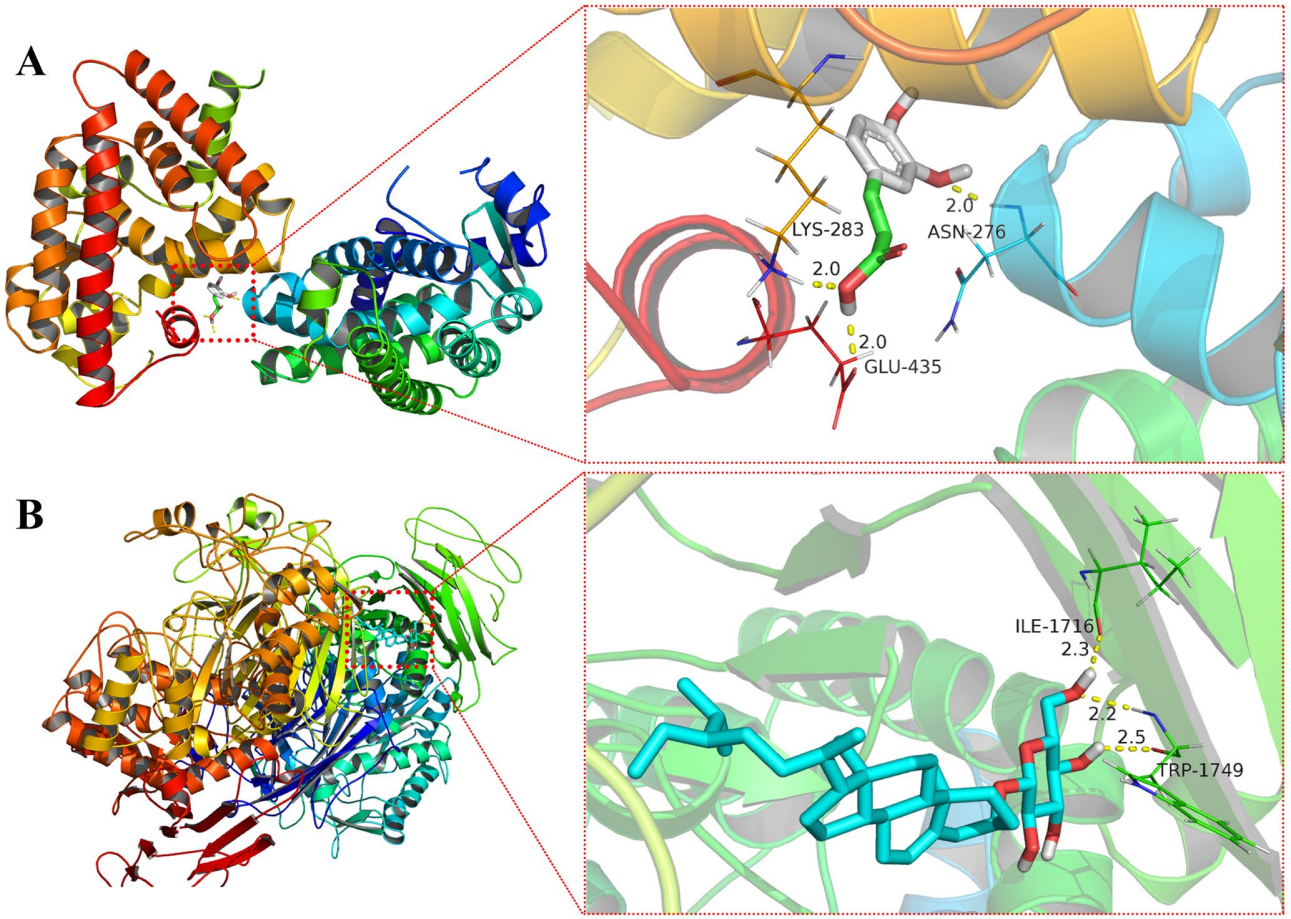
The visual analysis of molecular docking for caffeic acid with PPARD (A) and daucosterol with MGAM (B). The structures of targets were in the cartoon model with active component in the stick model and amino acid residues in the line model (the number meant the site in the protein). The yellow dashed lines with the number represented the distance (Å) between the ingredient and the amino acid residue.

In the molecular docking, it is generally accepted that a binding energy less than −5.0 kcal/mol indicates good binding activity between ingredient and target, and less than −7.0 kcal/mol means strong interaction force. The lower the binding energy, the stronger the docking activities [5, 20]. The PPAR signalling pathway plays an important role in alleviating oxidative damage [30, 31]. As shown in Table 4, the core hypoglycemic targets (PPARG and PPARD) and three related hypoglycemic ingredients (caffeic acid, ethyl linoleate and methyl linoleate) were selected for molecular docking. The results showed that the binding energies of caffeic acid and methyl linoleate with PPARG (PDB ID: 6TSG) and PPARD (PDB ID: 6A6P) were less than −5 kcal/mol except for ethyl linoleate to PPARG. Among these, caffeic acid exhibited the strongest docking effect to the PPARD receptor. As shown in Figure 6A, caffeic acid bound to PPARD through hydrogen bonds with asparagine (ASN), lysine (LYS) and glutamic acid (GLU, 2.0 Å) with a binding affinity of −6.8 kcal/mol.

**TABLE 4:** The molecular docking results of ingredients and targets.

Signalling Pathway	Targets	PDB ID	Ingredients	Affinity (kcal/mol)
PPAR signalling pathway	PPARD	6A6P	Caffeic acid	−6.8
			Ethyl linoleate	−6.5
			Methyl linoleate	−6.1
	PPARG	6TSG	Caffeic acid	−6.5
			Ethyl linoleate	−4.5
			Methyl linoleate	−5.8
Starch and sucrose metabolism Galactose metabolism	MGAM	3TOP	Daucosterol	−8.6
			Stigmasterol glucoside	−7.9
	GAA	5NN5	Daucosterol	−8.1
			Stigmasterol glucoside	−7.9

The results of molecular docking and the antioxidant activity indicated that the hypoglycemic effect of the alcoholic extract could be partly attributed to its antioxidant activity and the related PPAR signalling pathway.

#### 3.4.3 Enzymatic Activity

**FIGURE 7:**
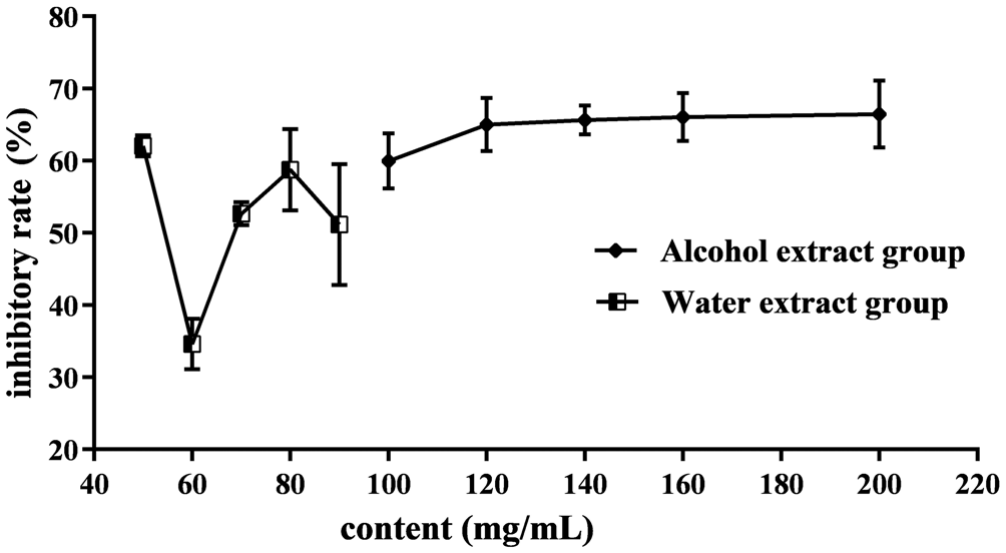
The inhibition activity of Lonicerae Japonicae Flos for α‐amylase (mean ± standard error, *n* = 3).

Intestinal maltase‐glucoamylase (MGAM), a promising therapeutic target for diabetes, catalyses the hydrolysis of 1,4‐α‐linked oligosaccharide substrates, thus playing an important role in carbohydrate digestion and subsequent glucose generation [32]. As listed in Table 4, both daucosterol and stigmasterol glucoside were evaluated for the inhibitory activities on MGAM (PDB ID: 3TOP) and Lysosomal alpha‐glucosidase (GAA, PDB ID: 5NN5). As shown in Figure 6B, daucosterol exhibited strong binding affinity for MGAM (−8.6 kcal/mol), forming hydrogen bonds with isoleucine (ILE, 2.3 Å) and two distinct interactions with TRP at distances of 2.2 and 2.5 Å. The results of molecular docking illustrated that the LJF ingredients had strong binding activity to the targets in the starch and sucrose metabolism, and the galactose metabolism. The inhibitory effect of the LJF extract was verified with the α‐amylase. As indicated in Figure 7, the alcohol extract displayed high inhibitory activity on α‐amylase in a dose‐dependent manner, with a maximum efficacy of 66.6% ± 4.6%. In contrast, the water extract did not exhibit obvious pattern on inhibition activity. It can be conferred that the different bioactivities of LJF extracts were probably stemmed from the solubility characteristics of its active components. As listed in Table S1, both daucosterol and stigmasterol glucoside, the key hypoglycemic compounds from LJG, had low water solubility. These observations align with previous findings that two alcohol‐soluble compounds from LJF (Lonjaponspiroside A and B) showed potent α‐glucosidase inhibitory activity [20]. To sum up, based on our experimental results, it could be concluded that the alcohol‐soluble components of LJF possess superior α‐amylase inhibitory and hypoglycemic activities compared to water‐soluble components.

## 4 Discussion

As a prominent public health issue currently, diabetes has prompted an urgent need of effective drugs. With a long history of practical applications on diabetes, LJF, a well‐known traditional Chinese medicine, has received much attention on its hypoglycemic effect recently. Although previous studies have confirmed the hypoglycemic effect of LJF, the hypoglycemic active ingredients and pharmacological mechanisms of LJF are not well understood. In this study, network pharmacology was employed to comprehensively screen the active ingredients, targets and signalling pathways for LJF on diabetes.

The hypoglycemic effect of LJF was a synergistic mechanism of multiple ingredients, multiple targets and multiple pathways. This study showed that these pathways, such as the PPAR signalling pathway, Galactose metabolism and Starch and sucrose metabolism, may be the main mechanisms for LJF on diabetes.

Oxidative damage is closely associated with high glucose stress, mitochondrial dysfunction and insulin resistance in diabetes [3, 31, 33, 34]. The PPAR signalling pathway plays an important role in alleviating oxidative damage [30, 31]. Song F et al. found acacetin exhibited a cardioprotective effect through upregulation of PPAR‐α and pAMPK, which in turn suppressed oxidative stress [34]. Pedro Góes Mesquita identified two endophytic fungi which could exhibit antioxidant activity and activate the PPAR receptors in promoting glucose homeostasis in diabetes [30]. As widely known, phenolic acids exhibit beneficial healthcare and nutritional value due to their good antioxidant capacity. The main active ingredients of LJF, caffeic acid derivatives, such as chlorogenic acid and isochlorogenic acid A‐C, have a highly conjugated system with multiple hydroxyl groups [13]. This molecular structure characteristic makes these ingredients effective electron or hydrogen atom donors in the process of eliminating reactive oxygen species. Therefore, LJF showed favourable antioxidative activities [13]. Wang D et al. found LJF could ameliorate the oxidative stress of diabetic rats [6]. Guo C et al. revealed LJF and chlorogenic acid could activate the AMPK/PPARα axes to modulate blood glucose [19]. On the other hand, the RAR signalling pathway, a key member of the nuclear receptor superfamily, regulates gene expression to influence cell differentiation, metabolism and inflammation. Emerging evidence links RAR signalling to diabetes, particularly type 2 diabetes. By activating the RAR/RXR (Retinoid X receptor) heterodimer, retinoic acid could regulate adipocyte differentiation, thereby influencing the expression of insulin signalling‐related genes, including PPARγ and GLUT4. Retinoic acid can also inhibit adipose tissue inflammation, thereby improving insulin resistance [35, 36]. In addition, retinoic acid can regulate glucose and lipid metabolism by inhibiting hepatic inflammatory response and inhibiting hepatic lipid accumulation [37, 38].

As an efficient target of diabetes, MGAM could hydrolyze the 1,4‐alpha linked oligosaccharide substrates [32], and α‐amylase plays an important role in the digestion and absorption of starch, which is the main source of blood glucose. The results of molecular docking and α‐amylase inhibition activity indicated the alcoholic extract could exert hypoglycemic effect by inhibiting the activity of amylase and delay or prolong the release time of glucose.

There are still some limitations in our study. Our findings support the medicinal value of LJF for treating diabetes, providing a valuable foundation for further research and development of LJF. However, it should be emphasized that the active ingredients require more validation, and the precise mechanisms by which LJF exerts its anti‐diabetic effects need more in‐depth investigation. Moreover, long‐term hyperglycemia would cause liver lesions and diabetic nephropathy, and our study showed the LJF extract exhibited a certain extent of protection on the hyperglycemia‐induced liver and kidney lesions. However, our current results were insufficiently detailed and convincing. We would carry out further study to support our viewpoint in the following study. Though metformin could decrease the level of MDA and increase the SOD level (Figure 5), there was no significant difference between the positive group and the model group (*p* > 0.05). It has been reported that the hypoglycemic mechanism of metformin primarily involves inhibiting hepatic gluconeogenesis, improving insulin sensitivity, and reducing intestinal glucose absorption [39], and the mechanism of metformin on MDA and SOD needs to be further explored.

## 5 Conclusion

This study employed an integrated strategy combining network pharmacology, molecular docking, and experimental validation to investigate the anti‐diabetic constituents, mechanisms, and evaluate the hypoglycemic effect of LJF. Network pharmacology successfully screened 73 active ingredients, 65 potential targets and 20 main signalling pathways involved in its hypoglycemic effects. The results of molecular docking, the PPI network and the ingredient‐target‐pathway network showed that 4 targets and three signalling pathways may play an essential role for LJF on diabetes. Animal experiments demonstrated that the LJF alcohol extract significantly decrease blood glucose levels compared to the model group (*p* < 0.05) and attenuated diabetes‐associated weight loss (*p* < 0.05). In addition, the LJF alcohol extract showed potent antioxidant activity and α‐amylase inhibitory activity. Collectively, these findings elucidate the multi‐component, multi‐target mechanisms of LJF on diabetic and provide a scientific basis for its therapeutic application.

## Author Contributions

**Guo Chao:** conceptualization, formal analysis, validation. **Li Chunyan:** resources, formal analysis, validation, funding acquisition. **Liu Xiaolong:** formal analysis, investigation. **Ren Lu:** writing – original draft, formal analysis. **Zhang Nan:** conceptualization, writing – review and editing, formal analysis, funding acquisition. **Xue Jintao:** conceptualization, writing – original draft, writing – review and editing, validation, formal analysis, methodology, software.

## Funding

This work was supported by the [University Key Research Projects of Henan Province] under Grant [number 25B360004, 24B360013, 23A360014], the [Natural Science Foundation of Henan Province] under Grant [number 252300423839], the [Backbone Teachers Program of Sanquan College of Xinxiang Medical University (North Henan Medical University)] under Grant [number SQ2025GGJS08], the [Key Scientific Research Projects of Higher Education Institutions in Henan Province] under Grant [number 23A350005], the [Key Research and Development Program of Henan] under Grant [number 261111314600] and the [Key Laboratory of Asymmetric Synthesis and Chiral Technology of Sichuan Province] under Grant [number 2024KFKT02].

## Ethics Statement

The animal experiment was approved by Animal Ethics Committee of Henan Medical University (No. XYLL‐20220169), and followed the internationally accepted principles for laboratory animal use and care, such as the European Community guidelines (EEC Directive of 1986; 86/609/EEC) and Guidelines for the Euthanasia of Animals (2020).

## Conflicts of Interest

The authors declare no conflicts of interest.

## Supporting information


**Figure S1:** The flowchart of network pharmacology.

**Figure S2:** The intersection analysis between the targets of ingredients and diabetes.

**Figure S3:** Islet morphology in control non‐diabetic group (A) and diabetic model group (B). Pancreatic islets are delineated by blue lines (H&E staining).

**Table S1:** The information of active ingredients in Lonicerae Japonicae Flos.

**Table S2:** The Gene Ontology (GO) enrichment analysis and the Kyoto Encyclopedia of Genes and Genomes (KEGG) enrichment analysis for hypoglycemic targets of Lonicerae Japonicae Flos.

## Data Availability

The original data of this study is included in Supporting Information.
