# Supplementary figures and images for: A Network Pharmacology and Experimental Validation Strategy for Hypoglycemic Study of Lonicerae Japonicae Flos on Diabetes
Source: J Cell Mol Med. 2026 May 2;30(9):e71167. doi: 10.1111/jcmm.71167 (PMC13135239; doi:10.1111/jcmm.71167)

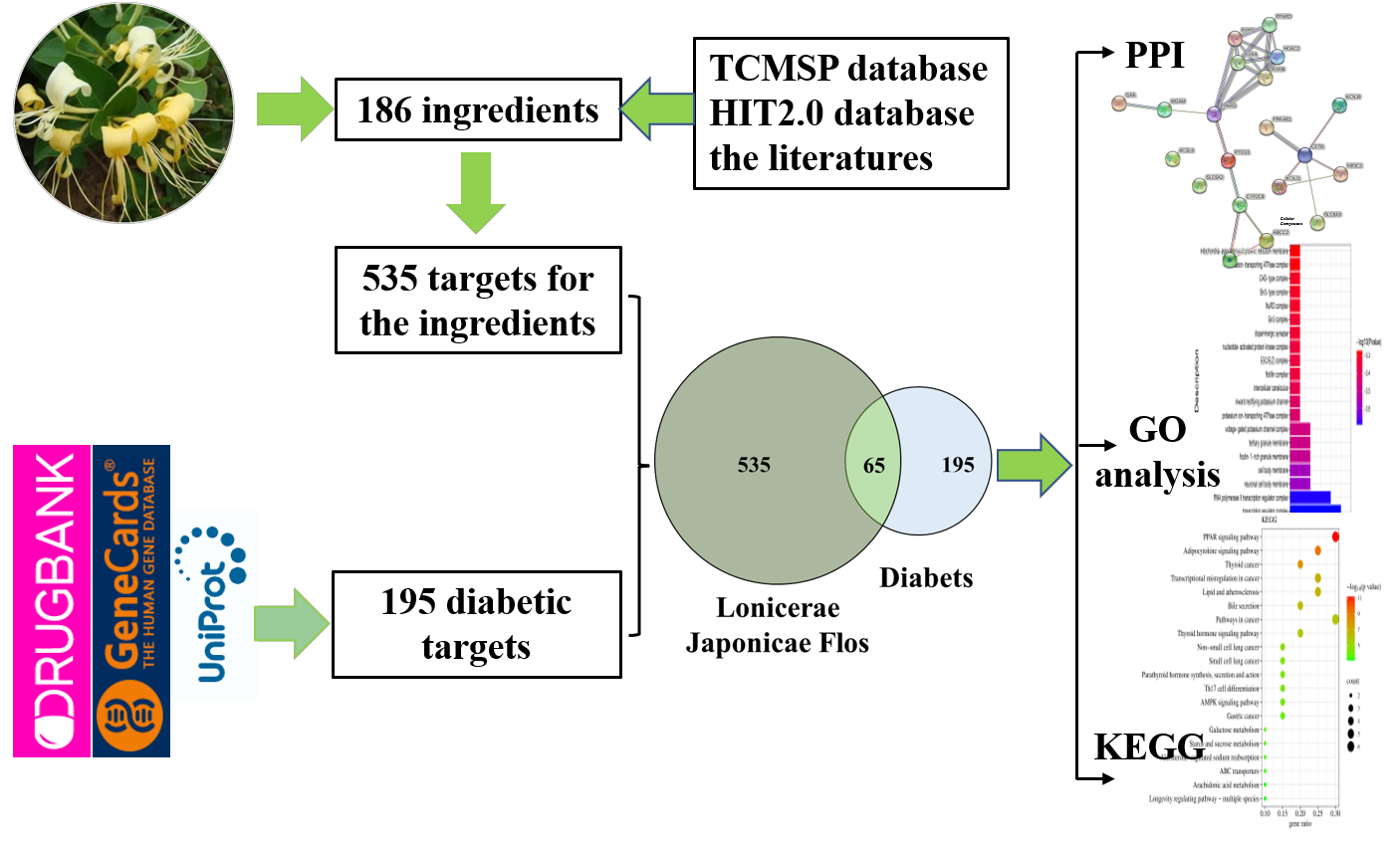

Supplement: Supplementary file 1 — Figure S1: The flowchart of network pharmacology. [file JCMM-30-e71167-s004.tif]

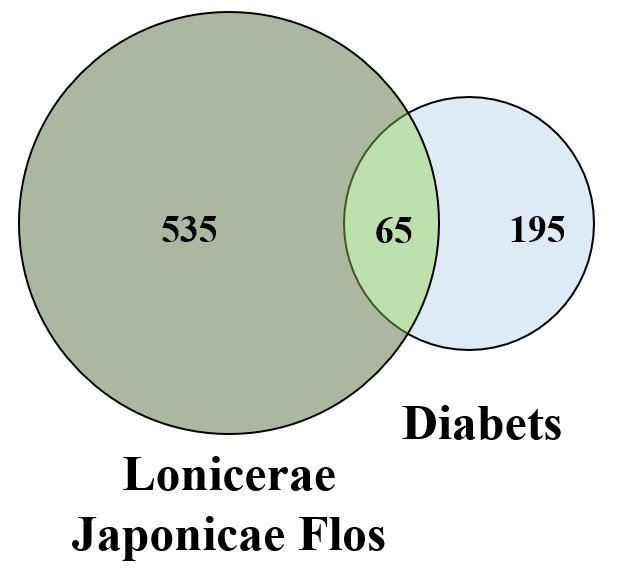

Supplement: Supplementary file 2 — Figure S2: The intersection analysis between the targets of ingredients and diabetes. [file JCMM-30-e71167-s003.tif]
